# An Update on the Sociomicrobiology of Quorum Sensing in Gram-Negative Biofilm Development

**DOI:** 10.3390/pathogens6040051

**Published:** 2017-10-21

**Authors:** Daniel Passos da Silva, Melissa C. Schofield, Matthew R. Parsek, Boo Shan Tseng

**Affiliations:** 1 Department of Microbiology, University of Washington School of Medicine, Box 357735, 1959 NE Pacific Street, Seattle, WA 98195-7242, USA; passosds@uw.edu; 2 School of Life Sciences, University of Nevada, Las Vegas Box MS 454004 4505 S. Maryland Parkway, Las Vegas, NV 89154, USA; schofi38@unlv.nevada.edu

**Keywords:** sociomicrobiology, quorum sensing, biofilm, signaling

## Abstract

Bacteria are social creatures that are able to interact and coordinate behaviors with each other in a multitude of ways. The study of such group behaviors in microbes was coined “sociomicrobiology” in 2005. Two such group behaviors in bacteria are quorum sensing (QS) and biofilm formation. At a very basic level, QS is the ability to sense bacterial density via cell-to-cell signaling using self-produced signals called autoinducers, and biofilms are aggregates of cells that are attached to one another via a self-produced, extracellular matrix. Since cells in biofilm aggregates are in close proximity, biofilms represent an ecologically relevant environment for QS. While QS is known to affect biofilm formation in both Gram-negative and Gram-positive species, in this review, we will focus exclusively on Gram-negative bacteria, with an emphasis on *Pseudomonas aeruginosa*. We will begin by describing QS systems in *P. aeruginosa* and how they affect *P. aeruginosa* biofilm formation. We then expand our review to other Gram-negative bacteria and conclude with interesting questions with regard to the effect of biofilms on QS.

## 1. Quorum Sensing in *Pseudomonas aeruginosa*

Bacteria are social creatures that interact and coordinate behaviors with each other in a multitude of ways. One mechanism involves cell-to-cell signaling, also known as quorum sensing (QS). QS is commonly found in both Gram-negative and Gram-positive species, and utilizes self-produced signals. These signals come in a variety of forms, e.g. small peptides, acyl-homoserine lactones (AHL), and quinolones [1,2,3,4]. Currently, there are a wide range of QS systems that have been identified, but AHL-based QS represents one of the most thoroughly studied systems [5,6].

The basic AHL-based QS system usually consists of three components: a cytoplasmic AHL synthase protein of the LuxI family, an AHL-responsive DNA-binding transcriptional regulator belonging to the LuxR family, and an acyl-homoserine lactone signal, which has a conserved homoserine lactone ring linked by an amide bond to side chains that vary depending upon the species and the system [1,4]. In canonical QS systems, the signal synthase and the transcriptional regulator are located on the chromosome in tandem or are separated by one or two genes [7]. In some species, there are *luxR* homologs that are not in the vicinity of a *luxI*-like gene, which are referred to as solos or orphans. 

Cells produce and release AHL signals at a low basal rate. AHL signals are capable of freely diffusing across the cell membrane, eliminating the need for a specific AHL-signal receptor on the cell surface. When the signals reach a critical threshold concentration in the local environment, they interact with the LuxR-family transcriptional regulator, which in turn changes gene expression by binding to a conserved DNA sequence in the promoter region (called a *lux* box). In several systems, the gene encoding the LuxI signal synthase is positively regulated by QS, leading to positive feedback of the system [8,9].

In *Pseudomonas aeruginosa,* there are two QS systems: *las* and *rhl*. The *las* system is composed of LasI, which synthesizes *N*-3-oxo-dodecanoyl-l-homoserine lactone, and LasR, the cognate transcriptional regulator that senses the signal. In the *rhl* system, RhlI is responsible for the production of *N*-butanoyl-l-homoserine lactone, which is recognized by RhlR. In addition, there is an “orphan” LuxR regulator called QscR that has been shown to bind the signal produced by LasI and to affect both the *las* and *rhl* systems. As there are *las*-boxes upstream of both *lasI* and *rhlI*, LasR not only positively auto-regulates the *las* system, but also promotes early activation of the *rhl* system. Strains lacking a functional *las* system where *rhl* is still functional have been reported, but *rhl* activation is delayed [10,11]. 

One relevant non-AHL QS system in *P. aeruginosa* is the *pqs* system. This system uses 2-alkyl-4-quinolones (AQs) as signals, and expression studies have shown that it has a relationship with the *las* and *rhl* regulons. The system is composed of the *pqsABCDE* operon, and three other transcriptional units, *pqsR* (also known as *mvfR), pqsH,* and *pqsL. P. aeruginosa* produces dozens of AQs and the most commonly associated with QS are 2-heptyl-3-hydroxy-4-quinolone (PQS) and its precursor 2-heptyl-4-hydroxyquinoline (HHQ). The synthesis of these molecules is dependent on the *pqsABCDE* operon, but production of PQS also requires *pqsH* under aerobic conditions via the oxidation of HHQ. Like AHL QS systems, *pqs* is subject to positive autoregulation via its LysR-family transcriptional regulator PqsR, which is known to regulate *pqsABCDE*-*phnAB* expression. 

QS in *P. aeruginosa* is very complex and can be modulated by transcriptional regulators (RsaL and MvaT), sigma factors (RpoS and RpoN), post-transcriptional regulators (RsmA), and even other QS systems (*pqs* system) [3,8,9,12,13,14,15,16]. Detailing all these levels of regulation is not within the scope of this review. The genes regulated by QS in *P. aeruginosa* have been studied by multiple groups using diverse methodologies and culturing conditions. Depending upon the study, the QS regulon usually comprises over 300 genes, of which about a hundred are common between the different studies. This suggests that the QS regulon is fairly plastic, changing with environmental conditions. Nevertheless, considering that the PAO1 genome has 5688 genes, over 5% of its genes are either directly or indirectly QS controlled, emphasizing the global regulatory nature of QS. Among the functions regulated by the *las* and *rhl* systems are several virulence factors that have been associated with biofilm formation, such as lectins, rhamnolipids, and siderophores [17,18,19].

## 2. QS-Regulated Factors that Influence Biofilm Formation in *P. aeruginosa*

**Figure 1:**
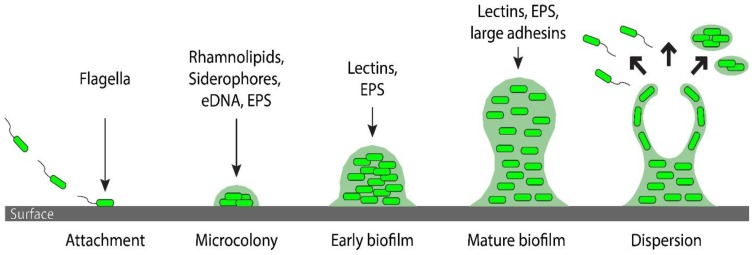
**Schematic of the stages of biofilm development regulated by quorum sensing factors.** The first stage of biofilm formation is the attachment of bacteria to a surface, which has been associated with flagella. In the second stage, the ability to produce rhamnolipids (swarming), siderophores (iron availability), eDNA and EPS (matrix formation) are thought to be essential for microcolony formation. In the subsequent stages of biofilm maturation, lectins, adhesins and EPS are important for the proper building of the matrix and localization of its components. The final stage is biofilm dispersion. Little mechanistic data is currently available to establish a role for quorum sensing in this process.

The first report to link *P. aeruginosa* QS to biofilm formation showed that, contrary to the large aggregates characteristic of wild-type biofilms, the *lasI* mutant produced a biofilm that was flat, structurally homogenous, and had a densely packed multilayer of cells that was sensitive to detergent treatment [20]. Additionally, a later study showed that furanones, which are AHL analogues that compete for the binding to the LuxR homologs, impacted biofilm formation producing a phenotype similar to that of a *lasI* mutant [21]. Subsequent studies attempted to identify which QS-controlled factors were responsible for the observed biofilm defect. These studies revealed that a wide range of QS-controlled functions are capable of influencing biofilm formation (Figure 1). 

*Las- and Rhl-controlled factors*. Rhamnolipid production, which is under the control of the *rhl* QS system, was shown to be required for maintaining the open spaces between biofilm aggregates. Rhamnolipids also affect a type of surface motility called swarming that has been implicated in biofilm development [22,23]. Two QS-dependent carbohydrate-binding lectins, LecA and LecB, were shown to influence biofilm formation, although the mechanism is unclear. Mutant strains were able to initiate biofilm formation by adhering to surfaces, but were not able to fully develop a mature biofilm [24,25]. Another QS-controlled factor is iron siderophores, such as pyoverdine. Mutants unable to produce this iron chelator were not able to generate biofilm aggregates [26].

*PQS-controlled factors.* Notably, the PQS system controls the expression of biofilm relevant functions that overlap with *las and rhl*-regulated elements, such as LecA [27] and siderophore production [28]. A unique PQS-controlled factor involved in biofilm formation is a component of the biofilm matrix: extracellular DNA (eDNA). In biofilm formation, the *pqs* QS system is responsible for an increased production of eDNA, which is capable of interacting with a positively charged exopolysaccharide present in the matrix (Pel). This interaction is thought to be important in generating the initial scaffolding for the biofilm. In strains lacking the ability to make PQS, mature biofilm aggregates never fully develop [27,29,30,31]. Thus, several QS-regulated functions have the potential to influence biofilm production (Figure 1). 

## 3. Environmental Parameters Influencing QS Control of Biofilm Formation

Considering that the QS regulon is fluid and changes in response to environmental conditions, it is not surprising that the importance of QS for biofilm formation can vary tremendously. Therefore, it is not uncommon to find conflicting reports in the literature regarding the importance of a given QS-controlled function for biofilm production. 

*Nutrition.* In *P. aeruginosa*, the carbon source can have a dramatic effect on the contribution of QS to biofilm formation. When grown on glutamate or glucose as the sole carbon source, QS mutants produce biofilms similar to those of the wild-type strain. However, when succinate is used as the sole carbon source, QS mutants produce biofilms that are structurally distinct from that of wild type. The authors attributed this phenotype to nutritionally-dependent QS control of swarming [32].

*pH.* QS is impacted by several non-biological elements of the environment. One such element is pH. AHLs are fairly stable under neutral and acidic pH, but at high pH, the half-life of these molecules can be reduced to minutes, due to chemical hydrolysis of the homoserine lactone ring [33,34]. Since the physiological activity of microbes can generate significant pH gradients from the exterior to the interior of biofilm aggregates [35], the microbes in the biofilm may influence and create QS signal concentration gradients. 

*Mass transfer.* Physical parameters may also be important; the impact of flow rates on QS signaling within a biofilm has been demonstrated [36,37]. QS relies on local concentrations of AHLs that freely diffuse across the membrane and into the environment. With high flow rates, the rate of signal accumulation inside the biofilm structure could vary due to signal wash-out [38]. As mass transfer would prevent the build-up of signal to inducing concentrations, this could potentially lead to wild-type and QS mutant strains being nearly indistinguishable. Conversely, at extremely low flow rates, diffusion of AHLs would be very low, creating pockets of high AHL concentrations within the community [39]. Finally, flow rates could influence the onset of QS, with high flow delaying initially attached cells and small cell aggregates from sensing quorum.

Much of the discussion associated with these points emphasize the contextual nature of QS and its importance in biofilm communities. For instance, *P. aeruginosa* can produce radically different biofilm infections on different surfaces. Biofilms produced in the airways of people suffering from cystic fibrosis are found in thick, static mucus. The bacteria obtain their nutrients from host-derived compounds that are released due to the damage caused by the bacteria. These aggregates are suspended, and are subject to little or almost no flow. On the other hand, *P. aeruginosa* can also colonize urinary catheters, causing urinary tract infections. These biofilms would experience a completely different environment with regard to the attachment surface and flow. One can imagine that the point at which QS initiates and its contribution to biofilm development could be completely different in these two types of infection.

## 4. QS-Regulated Factors that Influence Biofilm Formation in Other Species

QS affects biofilm formation in a myriad of species besides *P. aeruginosa* [40,41,42,43]. In many cases, it is not mechanistically clear how QS impacts biofilm structure. Here, we have focused on those non-Pseudomonad systems for which we have some mechanistic insight.

*Attachment and flagellar-based motility.* For some species, QS has been shown to regulate flagellar synthesis and activity, which influence the attachment of bacteria to a surface (Figure 1) [44]. In *Escherichia coli*, *mqsR* expression is stimulated in the presence of autoinducer-2 (AI-2), a QS signal that is produced by a LuxS-family synthase protein in many bacteria, but not in *P. aeruginosa*. MqsR positively regulates *qseB*, *fliA*, and *motA* [45], which are involved in flagellar gene expression [46], biosynthesis [47], and motor function [48], respectively. Strains bearing mutations in these flagellum-related genes, as well as *motB* and *fliF*, are defective in attachment to many types of surfaces [49]. In addition, the *fliA* and *fliF* genes have also been shown to be upregulated 10-fold and 12-fold, respectively, in wild type *E. coli* strains versus a LuxS mutant. These LuxS mutant strains produce significantly less flagellin and, consequently, fewer flagella [50]. QS has also been shown to regulate flagellar-based motility in *Vibrio vulnificus*. An *smcR* (a *luxR* homolog) mutant strain displayed a reduced swimming diameter compared to that of wild type [51]. 

In contrast, QS affects biofilm formation by negatively regulating flagellar-based motility in other species. Mutations in the *luxR* homolog *opaR* of *Vibrio parahaemolyticus* result in the production of significantly more lateral flagella, and are hypermotile compared to wild type strains [52]. Mutations within the *Burkholderia thailandensis luxR* homolog *btaR1* exhibit a similar hypermotility phenotype as *V. parahaemolyticus opaR* mutant strains [53,54]. However, *B. thailandensis btaR1* mutant cells show no difference in attachment relative to wild type [55], while a *V. parahaemolyticus opaR* mutants are attachment deficient [56]. Therefore, the role of QS in regulating motility and surface attachment is highly variable across species.

*Formation of mature biofilms.* In addition to attachment, QS regulates other aspects of biofilm formation, including accumulation of biofilm biomass, biofilm structure, and the dispersal of biofilm cells. *Burkholderia pseudomallei* requires a functional *bpsR* and *bpsI* (*luxR* and *luxI* homologues) for biofilm production; strains harboring mutations in these genes produced biofilms with approximately a 50% and 75% reduction in biomass, respectively, relative to wild type biofilms [57]. Similar to *B. pseudomallei*, mutations in genes required for AI-2 uptake, *lsrR* and *lsrK*, in *E. coli* result in a reduction of biofilm thickness and total biomass compared to wild type [58]. In addition, these mutant strains show a deficiency in autoaggregation [58]. QS has been shown to affect autoaggregation in *B. thailandensis* as well. QS system-1 (QS1) of *B. thailandensis* promotes autoaggregation [53], and mutant strains defective for QS1 formed biofilms with aberrant biofilm structure compared to wild type [55]. 

Similar to *B. pseudomallei*, *V. vulnificus* containing mutations in the *luxR* homolog *smcR* are deficient for biofilm formation on glass and polystyrene surfaces [51]. However, as previously discussed for *P. aeruginosa*, the contribution of QS to biofilm formation is environmentally dependent. Under minimal nutrient conditions, *smcR* mutant strains can form biofilms on polystyrene surfaces. In such conditions, the *smcR* mutant cells reached a maximum biomass sooner, and formed thicker biofilms than those of wild-type cells. At later stages, *smcR* mutant biofilms retained relatively high levels of biofilm biomass, suggesting that these strains exhibit a defect in biofilm dispersal compared to wild-type [59]. 

Similar to *P. aeruginosa*, QS can also regulate the production of biofilm matrix components, such as exopolysaccharides and cell surface proteins, in other organisms (Figure 1). In many *Vibrio* species, colony morphology correlates to exopolysaccharide production. Specifically, opaque colonies indicate an increased quantity of the exopolysaccharide Vps [42]. In *V. parahaemolyticus*, induction of the *luxR* homolog *opaR* produced opaque colonies from previously translucent strains [60]. Similarly, in *V. vulnificus*, mutant strains of the *luxR* homolog *smcR* displayed more translucent colonies than that of wild type, indicating a decrease in exopolysaccharide production [61]. This role of QS in biofilm matrix production is also seen in *E. coli* and *B. cenocepacia*. In *E. coli*, *ydgG* mutants (renamed *tqsA*), an exporter of AI-2 molecules, have increased levels of intracellular AI-2, and had approximately a four-fold increase in the production of curli fibers, which are important in adhesion and biofilm maturation [62]. In *B. cenocepacia*, QS positively regulates the cell surface protein BapA, which is required of normal biofilm formation [63]. Strains with a mutation in *bapA* formed biofilms characterized by separated discontinuous aggregates, with a biofilm structure that was more porous compared to the wild type biofilm [63].

*The curious case of Vibrio cholerae*. In contrast to the species mentioned above, *V. cholerae* presents a very different scenario in terms of the effect of QS regulation on biofilm formation. In the organisms mentioned above, high cell density conditions initiate QS-regulated functions that promote biofilm formation. However, this is not the case in *V. cholerae*. LuxO is a two-component response regulator in *V. cholerae* involved in the response to QS signals. Mutations in *luxO* are locked in a state mimicking high cell density. These mutants show an inability to form biofilms [2]. Additionally, mutations in the *luxR* homolog *hapR* that result in a state of mimicking low cell density conditions form larger pellicles than wild-type, and have been suggested to be deficient in biofilm dispersal [64]. Mutant strains of *hapR* also overproduce the exopolysaccharide Vps, and are defective in attachment and colonization in mouse models [64]. An organism that behaves in a similar fashion to *V. cholerae* is the Gram-positive pathogen *Staphylococcus aureus*. While QS is very different in *S. aureus* in comparison to the Gram-negative organisms described in this review, mutants for *agr*, a QS system in *S. aureus*, form biofilms with increased biomass in comparison to those of wild-type [65]. Additionally, similar to that suggested for *V. cholerae*, the *agr* system controls the expression of the *psm* genes, which encode surfactants that promote biofilm dispersal [66].

## 5. Future Questions/Directions

To date, much research has focused on the impact of QS on biofilm structure through the study of QS mutant strains. Since biofilm structure has been intimately linked to antimicrobial susceptibility, this has given rise to an interest in therapies directed towards QS as a way to treat biofilm infections. However, a number of interesting questions remain regarding the basic biology of QS and biofilm communities. 

*Moving beyond the impact of QS on biofilm structure.* For many species, QS controls the expression of secreted and/or extracellular functions. In many cases, the extracellular activity of these enzymes in the context of a single cell would be irrelevant. In the presence of a quorum of cells however, the enzyme levels and activity are sufficient to provide a benefit to the cells producing them. If a quorum is achieved in a planktonic community, the assumption is that this must represent, in some fashion, a closed system. In other words, these cells are present in a fixed volume that is not subject to the effects of mass transfer. Thus, it is assumed that secreted QS-controlled enzymes, and the QS signals that precede them, are present in a fixed concentration in the surrounding bulk fluid.

In the context of a biofilm, this point is superimposed on an additional layer of complexity. The stationary/sessile nature of the community might be influenced by external forces, such as fluid flow, that might remove QS-controlled enzymes from the biofilm. One simple inference may be that if QS signals can achieve a high enough concentration in the biofilm system, that QS-controlled extracellular functions may provide a transient benefit. However, much of the energy invested in producing these enzymes would be wasted as these enzymes would eventually be removed from the community by mass transfer. This begs the question as to whether there are mechanisms by which a biofilm community can selectively retain extracellular enzymes. There are many potential mechanisms by which this might occur, including specific interactions of these enzymes with biofilm exopolysaccharides.

Another simple question that remains largely unanswered is how QS can influence a biofilm community apart from structure? Depending upon the species, there are many different classes of QS-regulated functions. These might include functions involved in nutrient acquisition, protecting the community from environmental stressors, and antimicrobial activity. One can imagine that mutant strains defective for these functions may have a tremendous impact on the community that is independent of biofilm structure. How these functions would work in a structured system like a biofilm is an interesting question. 

*Ecology of cheating in structured systems.* An expanding area of research in QS draws upon classic ecological theory. The appearance and significance of cheater subpopulations within a QS community is one such question that has been investigated and has relevance to disease in some species. The concept of public and private goods is also central to many of the research directions in this area. However, most of this research has been conducted on planktonic populations of cells.

Considering how these concepts would play out in a biofilm is intriguing. What would be the distribution and frequency of cheaters in a biofilm system? What would be the carrying capacity of cheaters in such a system, and what parameters would influence this? As cheaters appear in the system, would they give rise to clonal pockets of cheaters whose size would be limited by the mass transfer efficiency of public goods? These points are important since in many cases the “real-world” significance of cheating would pertain to biofilm communities. For example, the ability to form biofilms is a feature of many chronic diseases that *P. aeruginosa* can cause. Additionally, since QS mutant strains are frequently isolated from these infections, the importance of QS cheaters has been hypothesized to be important as well. Thus, if QS cheating occurs in a biofilm, how it occurs and what influences it may be very important questions for the scientific community to address.
